# Continuous lengthening potential after four years of magnetically controlled spinal deformity correction in children with spinal muscular atrophy

**DOI:** 10.1038/s41598-020-79821-x

**Published:** 2020-12-30

**Authors:** Heiko M. Lorenz, Marina M. Hecker, Lena Braunschweig, Batoul Badwan, Konstantinos Tsaknakis, Anna K. Hell

**Affiliations:** Pediatric Orthopaedics, Department of Trauma, Orthopaedic and Plastic Surgery, University Medical Center Goettingen, Robert-Koch-Str. 40, 37075 Goettingen, Germany

**Keywords:** Anatomy, Medical research

## Abstract

Magnetically controlled growing rods (MCGR) are commonly implanted for the treatment of early-onset scoliosis. While most authors report favorable short-term results, little is known about long-term deformity correction. This prospective cohort study assesses spinal deformity control in a homogeneous spinal muscular atrophy (SMA) patient group treated with MCGR implants, a standardized lengthening protocol and a minimum follow-up of four years. 17 SMA patients with progressive scoliosis were treated with MCGR implanted parallel to the spine with rib-to-pelvis fixation. Radiologic measurements were performed before and after MCGR implantation and during external lengthening procedures. These included measurements of the scoliotic curve, kyphosis, lordosis, pelvic obliquity and the spinal length. Additional clinical data of the complications were also analyzed. 17 children (mean age 7.4 years) were surgically treated and underwent a total of 376 lengthenings. Complication rates were 3.5% in respect to all interventions or 41% of the patients had complications during 3.5% of the lengthening sessions. The initial implantation significantly reduced the main scoliotic curve by 59%, with the correction remaining constant throughout the follow-up. Pelvic obliquity was also significantly and permanently corrected by 72%, whereas kyphosis and lordosis were not influenced. The spinal length could be significantly increased mostly during the first year of treatment. Bilateral implantation of MCGRs for correction of spinal deformity in children with SMA showed no decrease of the lengthening potential during a four-year follow-up. Therefore, the previously described ‘law of diminishing returns’ could not be applied to this patient population.

Level of Evidence/Clinical relevance: Therapeutic Level IV.

## Introduction

Spinal muscular atrophy (SMA), a progressive neuromuscular disorder with loss of lower motor neurons and progressive muscle weakness, is caused in the majority of cases by a genetic defect in the survival motor neuron 1 (*SMN1*) gene^1^. The manifestation of the disease differs widely ranging from very severe (SMA type I) to mild (SMA type IV). Recently, SMA received increased attention due to new treatment options. The antisense oligonucleotide nusinersen increases the level of SMN protein through modulation of alternate splicing of the *SMN2* gene, thus functionally converting it into *SMN1*. In 2016, nusinersen received FDA approval for intrathecal injection in the USA. In May 2019, the FDA approved an *SMN1*-gene targeting therapy for newborn and children up to two years old (zolgensma, onasemnogene abeparvovec-xioi). The first oral medication, risdiplam, was approved in August 2020.

Despite the aforementioned treatment advances, the majority of children with SMA will develop spinal deformity^2^, the treatment of which still lacks options in severe cases. According to the 2018 update^2^ of the consensus statement of the 2007 International Conference on the Standard of Care of SMA, orthotic braces may be used in moderate deformity. In severe curves, however, lung impairment is a restricting factor^3,4^ for bracing, the later hardly having any effect on curve progression.

Growth-friendly spinal implants such as growing rods or vertical expandable prosthetic titanium ribs (VEPTR) have been proven effective in the treatment of severe deformity of the growing spine^5,6^. However, most of these require repetitive surgical lengthening procedures, which may result in anesthesiologic complications^7^, as well as implant related problems^8–10^. Even though spinal fusion has been performed in young children with SMA in the past^11^, it is currently not being favored by most authors^2^. Definite spinal fusion, however, still remains the treatment of choice for spinal deformity in adolescents with SMA^12^.

A surgical technique has been described for the treatment of young children with SMA^13^. An implantation of magnetically controlled growing rod (MCGR) devices in combination with a bilateral rib-to-pelvis fixation is followed by ambulatory lengthening sessions, eliminating the need for further surgical intervention. Favorable two-year results using this technique have already been reported^14^. However, the question has still to be answered, whether the effect of MCGR treatment diminishes in the long-term. Sankar et al.^10^ reported a reduced lengthening potential of growing rods over time, calling it the ‘law of diminishing returns’, while similar effects have been reported for MCGR^15,16^. The above studies are based on a heterogeneous population of early-onset scoliosis patients (e.g. idiopathic, congenital, syndromic or neuromuscular scoliosis) and therefore comparison of treatment effects is highly debatable.

To our best knowledge this paper conducts for the first time a prospective investigation with a minimum follow-up of four years or longer on a homogeneous group of children with SMA and spinal deformity, which were treated with bilateral MCGRs and rib-to-pelvis fixation. All children had the same diagnosis, received the same implant construct and followed the same lengthening protocol. By eliminating influencing factors, this unique study design allows us to examine the effect of magnetically controlled devices over time and to assess a possible diminishing lengthening potential in this group of patients.

## Material and methods

**Figure 1:**
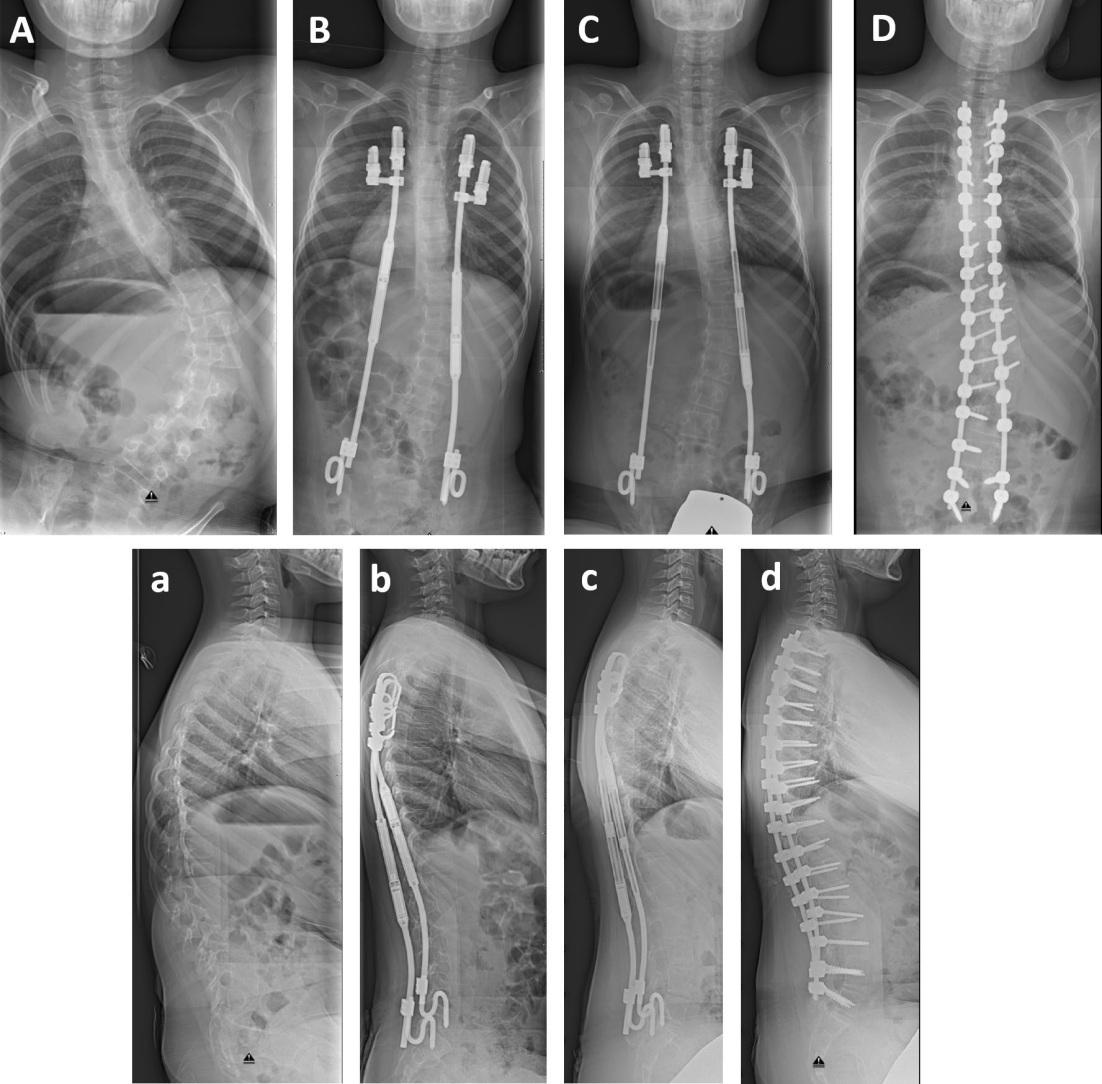
Posteroanterior (**A**–**D**) and lateral radiographs (a–d) of a nine-year old boy with SMA and spinal deformity. The scoliotic curve was corrected from 81° (**A**) to 27° (**B**) directly after MCGR implantation. This result could be maintained within the four-year follow-up (**C**) and after spondylodesis (**D**). The sagittal profile could be improved. Initial kyphosis of 42° (a) could be corrected to 29° directly after surgery (b) and to 36° during follow-up (c) and spondylodesis (35°, d).

Following approval of the institutional ethical review committee of the University Medical Center Goettingen, we performed a prospective study on 17 children diagnosed with SMA and progressive spinal deformity (Fig. 1A,a). The ethics committee waived the need for informed consent for the study. The participants were informed about the purpose of the study and informed consent was obtained for all patient images from a parent and/or legal guardian. All patients underwent implantation of MCGR implants with a bilateral fixation between ribs and pelvis (Fig. 1B,b,C,c) by HML, KT and AKH between 2012 and 2015 and were followed for a minimum of four years before spinal fusion (Fig. 1D,d). The first outpatient expansion procedure of 5 mm was carried out five months after surgical implantation, with following non-surgical lengthenings taking place every three months thereafter. After approximately two and a half years, surgical exchange of the MCGR was required at the point of maximal distraction of the implants.

Anterior–posterior and lateral radiographs in a sitting position were analyzed to assess the following parameters prior to and after initial MCGR implantation, as well as during the follow-up period of at least four years. Values for the main scoliotic curve, kyphosis and lordosis were obtained using the Cobb method^17^. Pelvic obliquity was assessed by measuring the angle between a horizontal line and the tangent of the iliac crests. Spinal length was defined as the distance between the sacrum and the center of T3, corresponding to the highest instrumented rib pair. All measurements were performed by two independent examiners (average deviation between the investigators was 0.042). Additional clinical data, such as application of nusinersen or the complication rate, were acquired.

The obtained data were reviewed statistically with a paired Student’s t-test using the Excel (Microsoft Corporations, Redmond, USA). All data are presented as mean ± standard error of the mean. Statistical significance between the values measured at different time points and the initial value was determined with levels as *p* < 0.05 (*), *p* < 0.01 (**) and as *p* < 0.001 (***).

### Ethical approval

All procedures performed in studies involving human participants were in accordance with the ethical standards of the institutional and/or national research committee and with the 1964 Helsinki declaration and its later amendments or comparable ethical standards.

## Results

Data of 17 children with SMA and scoliosis (8 females, 9 males) were analyzed before and after MCGR implantation and during following lengthening procedures (average 14.5 expansions within 4.5 years) (Table 1). All children were non-ambulatory, wheelchair dependent and with insufficient cephalic control, ranging from permanent to partial use of a headrest. Clinical presentation met the criteria of SMA type II.

**Table 1:** Patient demographics.

Variable	Value
**No. of patients**	
Females	17
Males	8
**Primary implantation (no. of patients)**	
VEPTR	2
MAGEC	15
**MAGEC treatment**	
Average age at MAGEC implantation in years (SD)	7.4 (± 1.6)
Number of lenghtenings (SD)	14.5 (± 2.1)
Duration in years (SD)	4.5 (± 0.5)
No. of rod exchanges	16
**No. of patients with nusinersen therapy (percentage)**	10 (59%)
Complications	13
Requiring surgical revision	8

Surgical implantation was carried out at a mean age of 7.4 years (SD = 1.6; range 5.0–9.4). Two children had prior to MCGR treatment, bilateral VEPTR rib to pelvis constructs for 2.2 and 3.2 years, respectively. During conversion surgeries from VEPTR to MCGR, these patients kept their initial fixation anchors. An overall of 376 interventions was performed within the follow-up period of 4.5 years (SD = 0.6; range 4.1–5.3): 15 initial surgeries, 2 conversion surgeries from VEPTR to MCGR, 343 non-surgical lengthening procedures and 16 MCGR exchange surgeries. Ten patients received nusinersen therapy during MCGR treatment (on average at 2.8 years after implantation), after its approval in Germany in 2017.

13 complications occurred during the follow up period, resulting in an overall complication rate of 3.5% (13 in 376 interventions), eight of them requiring surgical revision (Table 1). The index patient experienced implant dislocation because of different implant diameters, which was surgically corrected with the use of a connector. Rib fractures with implant dislocation led to revision surgery in three cases and superficial wound healing problems in two cases. Another two cases needed revision surgery after infection. Further complications could be managed conservatively. Overall, these 13 complications appeared in seven patients over time, which amounts to a patient-related complication rate of 41%.

### Main scoliotic curve

**Figure 2:**
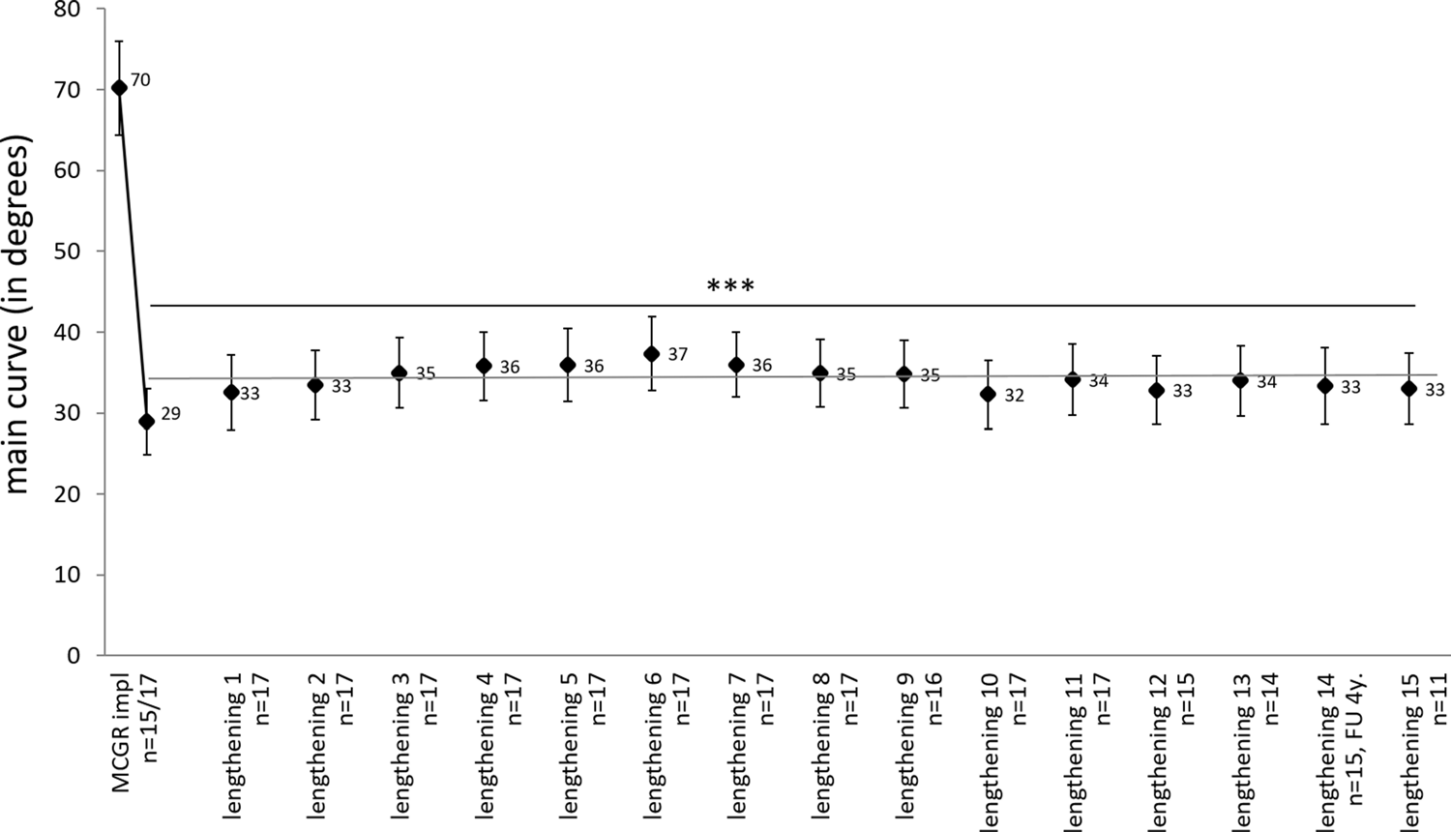
Development of the main scoliotic curve angle (mean ± standard error, in degrees) before and after MCGR implantation and after every lengthening procedure with n > 10. Significance (***p < 0.001) between the time points and the initial value was evaluated with paired Student’s t-test. The regression line is drawn for all values post implantation. Patients have a minimum follow-up of four years, but radiographs were not taken at each evaluation and therefore numbers differ.

At initial implantation of the MCGR system, the main thoracic or thoracolumbar curve was reduced by 59% from mean angles of 70° (SD = 19.1; range 35.4–97.4) to 29° (SD = 16.7; range 2.8–61.7) (Student’s t-test, *p* < 0.001) (Fig. 2). The overall achieved curve correction could be effectively maintained (*p* < 0.001) during the follow-up of 4.5 years. A regression line was drawn for all values post implantation with a slope of almost zero (0.0004).

### Pelvic obliquity

**Figure 3:**
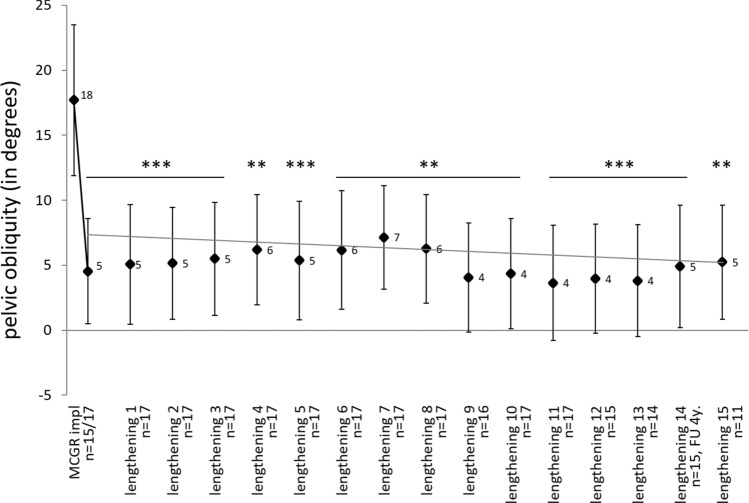
Development of the pelvic obliquity (mean ± standard error, in degrees) before and after MCGR implantation and after every lengthening procedure with n > 10. Significance (**p < 0.01, ***p < 0.001) between the time points and the initial value was evaluated with paired Student’s t-test. The regression line is drawn for all values post implantation.

Pelvic obliquity was also significantly reduced (72%) by magnetically controlled therapy from 18° (SD = 11.3; range 4–41) to 5° (SD = 2.9; range 1–10) after implantation (*p* < 0.001; Fig. 3). Pelvic obliquity remained on a low level throughout the whole follow-up period of 19 lengthening procedures (up to 5.3 years), ranging from 2° to 7°. The regression line drawn for all values post implantation shows a slight decrease within the follow-up period.

### Kyphosis and lordosis

Thoracic kyphosis measured 45° in the sagittal plane (SD = 13.1; range 31–67) before MCGR implantation and could be reduced to 34° (SD = 11.8; range 17–50) immediately upon implantation surgery (*p* = 0.017). In the following three procedures, kyphosis increased on average to 39° and could be kept stable from there on, with slight variations between 34° and 40°.

**Figure 4:**
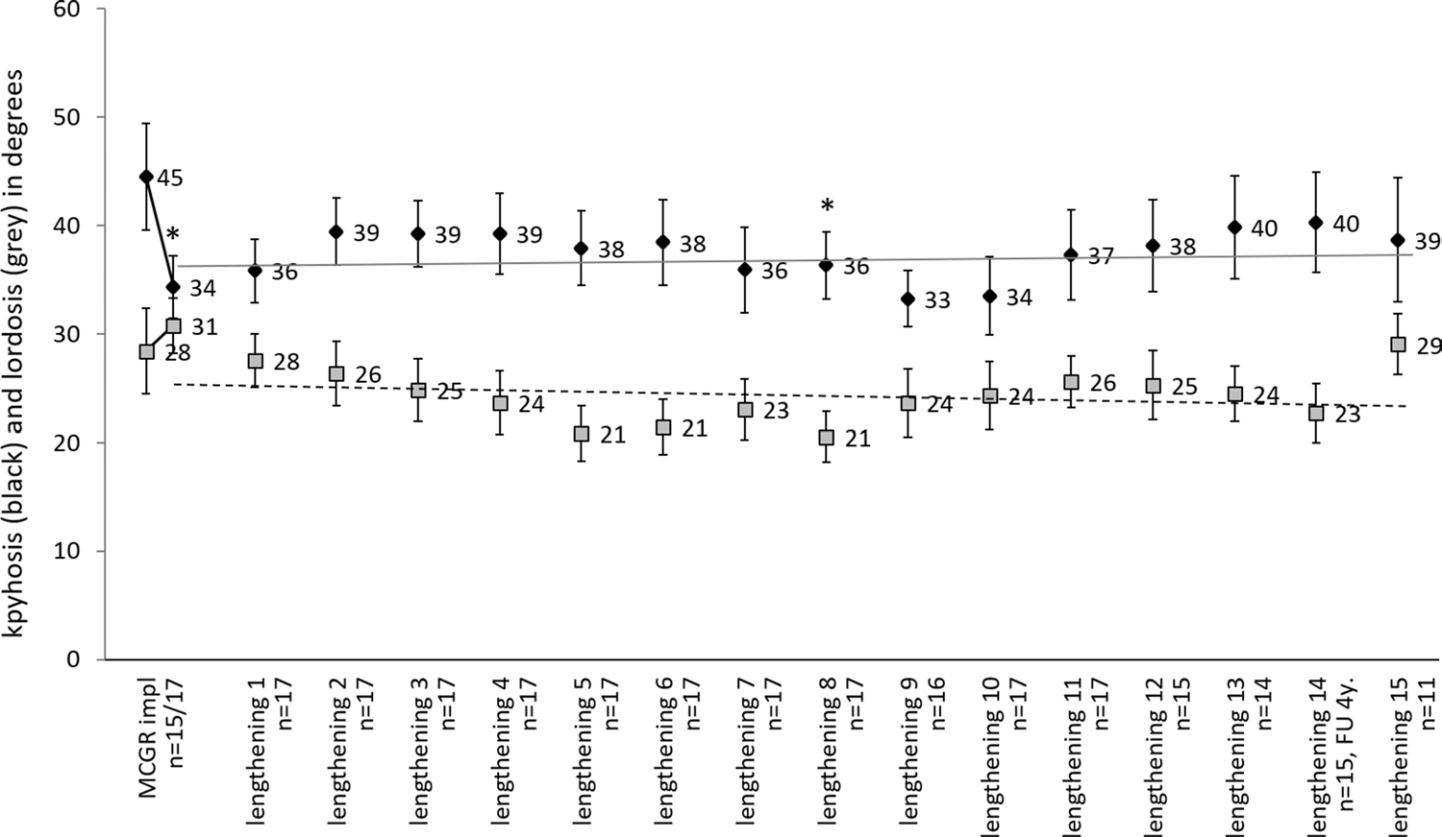
Development of thoracic kyphosis (black; mean ± standard error, in degrees) and lumbar lordosis (grey; mean ± standard error, in degrees) before and after MCGR implantation and after every lengthening procedure with n > 10. Significance (*p < 0.05) between the time points and the initial value was evaluated with paired Student’s t-test. The regression lines are drawn for all values post implantation.

Lumbar lordosis was not significantly affected by MCGR therapy, with average values between 21° and 31° (Fig. 4).

### Spinal length

**Figure 5:**
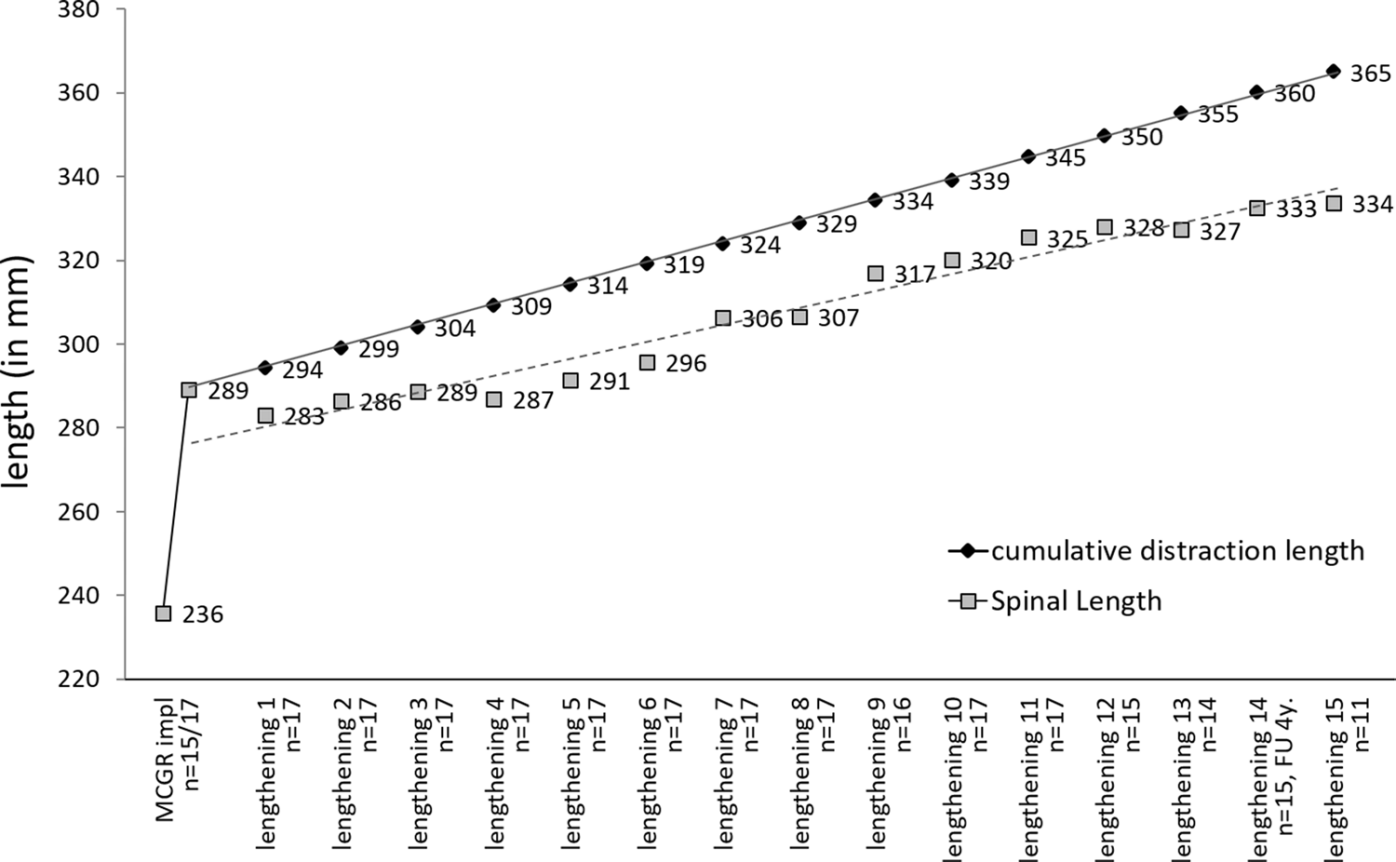
Development of the spinal length (grey; mean, in mm) and the cumulative distraction length (black; mean, in mm) before and after MCGR implantation and after lengthening procedures. The regression lines are drawn for all values post implantation.

The spinal length averaged 236 mm (SD = 24.2; range 195–270) before therapy and increased to 289 mm (SD = 18.6; range 256–316) directly after device implantation (*p* < 0.001; Fig. 5). The spinal length was stagnant during the first year after implantation despite continuous distraction, whereas it increased steadily over time in the course of further treatment. The calculated cumulative distraction length was slightly higher during the whole follow-up due to this first stagnant phase of spinal length. There was no diminishing effect on spinal length during the later follow-up years.

## Discussion

Severe spinal deformity at a young age is a very common and challenging problem in children with SMA. In recent years, the implantation of MCGR systems has become the treatment of choice, because of the advantage of fewer surgical interventions and complications^18^ compared to other implants^13,14^. Efficiency of MCGR implants was shown in several studies^18–20^.

However, long-term effects of MCGR treatment and a possible reduced lengthening potential over time are still under discussion^15,16^. This paper conducts for the first time to our knowledge a prospective investigation of a homogeneous patient population of children with SMA and spinal deformity, treated with MCGR and a follow-up of four years or longer.

In our patient group, MCGR implantation was able to sufficiently reduce the main scoliotic curve by 59%. This is slightly better than results in previous studies of MCGR implants, which report an initial surgical curve reduction potential between 33 and 41%^18,19,21^.

A standardized lengthening protocol of 5 mm every three months during a follow-up period of 4.5 years was able to maintain these favorable results. Interestingly, the increase in spinal length did not diminish over time. Therefore, the previously reported^10^ reduced long-term lengthening potential in growing rods does not seem to apply for children with SMA. Various studies analyzing the possibility of a diminishing lengthening effect of MCGR implants over longer follow-up periods are controversial. Ahmad et al.^15^ reported on a reduced lengthening potential after an average of 30 months and Studer et al.^16^ after an average of 25 months. On the contrary, Cheung et al.^22,23^ as well as Gardner^24^ found no ‘law of diminishing returns’ after 46, 50 and 24 months follow-up respectively.

Possible reasons for our findings may reflect a high flexibility of spinal deformity in SMA children with SMA, as well as short time intervals between lengthening procedures. Akbarnia et al.^25^ compared results of three versus six months distraction intervals after traditional growing rod implantation in early-onset scoliosis children. They found better outcomes in the short distraction group, which is in accordance to our findings for MCGRs. The shorter intervals most likely result in a more physiological spinal lengthening and even possible promotion of vertebral growth^26^.

The analysis of the data in our patients reveals a decrease of the spinal length between the index surgery and the first lengthening five months later. Manual distraction during surgery is probably able to apply higher pressure in comparison to externally applied MCGR distraction forces. Intraoperative manual distraction was able to apply up to 608 Nm^27^, while Rolton et al.^28^ cited a maximum force for externally controlled lengthening of MCGR systems of 270 Nm. Therefore, the initially higher force produced a maximum spinal length which was slightly reduced within the following months. Another reason for reduced spinal length might be implant migration into the pelvis and/or bending of ribs as a response to high implant pressure on bone stock.

Similar to scoliotic correction, pelvic obliquity was highly responsive to the MCGR treatment directly after surgery and over time. This corrective effect of bilateral paraspinal implants on pelvic obliquity has been described before^14,29^.

Implantation of MCGRs did not significantly influence thoracic kyphosis or lumbar lordosis in the sagittal plane over time. On average, normal values could be maintained during the treatment course.

Analyzing 376 interventions, there was an overall complication rate of 3.5%. Evaluating complications per patient, the investigated group of SMA patients had a complication rate of 41%, which was similar to previously described literature data which report of 40% to 57%^18,30^. In the literature^18,31,32^, the risk of developing complications increases with longer treatment periods. Therefore, an increased complication rate might be found at older age towards the cessation of growth and during longer follow-up.

Despite strong long-term data on spinal deformity control with MCGR systems in patients with SMA, our study is limited in respect to analysis of cranial junctional kyphosis. The majority of our SMA children had not normal cephalic control and thus limited standardized radiological imaging in a sitting position. Consequently, junctional kyphosis could not be analyzed. Additionally, the lack of the ‘law of diminishing returns’ in this homogeneous patient group may not be applied to other entities of early-onset scoliosis children.

## References

[1] Brzustowicz LM (1990). Genetic mapping of chronic childhood-onset spinal muscular atrophy to chromosome 5q1 1.2–13.3. Nature.

[2] Mercuri E (2018). Diagnosis and management of spinal muscular atrophy: Part 1: Recommendations for diagnosis, rehabilitation, orthopedic and nutritional care. Neuromuscul. Disord. NMD.

[3] Tangsrud SE, Carlsen KC, Lund-Petersen I, Carlsen KH (2001). Lung function measurements in young children with spinal muscle atrophy; a cross sectional survey on the effect of position and bracing. Arch. Dis. Child..

[4] Morillon S (2007). Effect of thoracic bracing on lung function in children with neuromuscular disease. Ann. Readapt. Med. Phys..

[5] Catteruccia M (2015). Orthopedic management of scoliosis by garches brace and spinal fusion in SMA type 2 children. J. Neuromuscul. Dis..

[6] Mesfin A, Sponseller PD, Leet AI (2012). Spinal muscular atrophy: Manifestations and management. J. Am. Acad. Orthop. Surg..

[7] Islander G (2013). Anesthesia and spinal muscle atrophy. Paediatr. Anaesth..

[8] Plaass C, Hasler CC, Heininger U, Studer D (2016). Bacterial colonization of VEPTR implants under repeated expansions in children with severe early onset spinal deformities. Eur. Spine J..

[9] Wagner L (2018). Detection of bacteria colonizing titanium spinal implants in children. Surg. Infect..

[10] Sankar WN (2011). Lengthening of dual growing rods and the law of diminishing returns. Spine.

[11] Klemme WR, Denis F, Winter RB, Lonstein JW, Koop SE (1997). Spinal instrumentation without fusion for progressive scoliosis in young children. J. Pediatr. Orthop..

[12] Hell AK (2020). Children with spinal muscular atrophy with prior growth-friendly spinal implants have better results after definite spinal fusion in comparison to untreated patients. Neurosurgery.

[13] Hell AK, Groenefeld K, Tsaknakis K, Braunschweig L, Lorenz HM (2018). Combining bilateral magnetically controlled implants inserted parallel to the spine with rib to pelvis fixation: surgical technique and early results. Clin. Spine Surg..

[14] Lorenz HM (2017). Magnetically controlled devices parallel to the spine in children with spinal muscular atrophy. JB JS Open Access.

[15] Ahmad A (2017). Quantifying the ‘law of diminishing returns’ in magnetically controlled growing rods. Bone Jt. J..

[16] Studer D, Heidt C, Büchler P, Hasler CC (2019). Treatment of early onset spinal deformities with magnetically controlled growing rods: a single centre experience of 30 cases. J. Child. Orthop..

[17] Cobb J (1948). Outline for the study of scoliosis. Am. Acad. Orthop. Surg..

[18] Lebon J (2017). Magnetically controlled growing rod in early onset scoliosis: a 30-case multicenter study. Eur. Spine J..

[19] Akbarnia BA (2013). Next generation of growth-sparing techniques: preliminary clinical results of a magnetically controlled growing rod in 14 patients with early-onset scoliosis. Spine.

[20] Hickey BA (2014). Early experience of MAGEC magnetic growing rods in the treatment of early onset scoliosis. Eur. Spine J..

[21] Dannawi Z, Altaf F, Harshavardhana NS, El Sebaie H, Noordeen H (2013). Early results of a remotely-operated magnetic growth rod in early-onset scoliosis. Bone Jt. J..

[22] Cheung JPY, Bow C, Samartzis D, Kwan K, Cheung KMC (2016). Frequent small distractions with a magnetically controlled growing rod for early-onset scoliosis and avoidance of the law of diminishing returns. J. Orthop. Surg. Hong Kong.

[23] Cheung JPY (2018). Rod lengthening with the magnetically controlled growing rod: Factors influencing rod slippage and reduced gains during distractions. Spine.

[24] Gardner A (2017). Does the law of diminishing returns apply to the lengthening of the MCGR rod in early onset scoliosis with reference to growth velocity?. J. Spine Surg. Hong Kong.

[25] Akbarnia BA (2008). Dual growing rod technique followed for three to eleven years until final fusion: The effect of frequency of lengthening. Spine.

[26] Yang S, Andras LM, Redding GJ, Skaggs DL (2016). Early-onset scoliosis: A review of history, current treatment, and future directions. Pediatrics.

[27] Noordeen HM (2011). In vivo distraction force and length measurements of growing rods: Which factors influence the ability to lengthen?. Spine.

[28] Rolton D, Thakar C, Wilson-MacDonald J, Nnadi C (2016). Radiological and clinical assessment of the distraction achieved with remotely expandable growing rods in early onset scoliosis. Eur. Spine J..

[29] Hasler C-C, Mehrkens A, Hefti F (2010). Efficacy and safety of VEPTR instrumentation for progressive spine deformities in young children without rib fusions. Eur. Spine J..

[30] Thakar C (2018). Systematic review of the complications associated with magnetically controlled growing rods for the treatment of early onset scoliosis. Eur. Spine J..

[31] Teoh KH (2016). Magnetic controlled growing rods for early-onset scoliosis: a 4-year follow-up. Spine J..

[32] Kwan KYH (2017). Unplanned reoperations in magnetically controlled growing rod surgery for early onset scoliosis with a minimum of two-year follow-up. Spine.

